# The mission characteristics of a newly implemented rural helicopter emergency medical service

**DOI:** 10.1186/s12873-018-0176-3

**Published:** 2018-08-29

**Authors:** Daniel Kornhall, Robert Näslund, Cecilia Klingberg, Regina Schiborr, Mikael Gellerfors

**Affiliations:** 1 Swedish Air Ambulance (SLA), Mora, Sweden; 2 East Anglian Air Ambulance, Cambridge, UK; 3 Nordland Hospital, Bodø, Norway; 4 Department of Anaesthesiology and Intensive Care, Falun County Hospital, Falun, Sweden; 5 Department of Anaesthesiology and Intensive Care, Mora Hospital, Mora, Sweden; 6 Department of Clinical Science and Education, Section of Anaesthesiology and Intensive Care, Karolinska Institutet, Stockholm, Sweden; 7 Department of Anaesthesiology and Intensive Care, Sodersjukhuset, Stockholm, Sweden; 8 SAE Medevac Helicopter, Swedish Armed Forces, Linkoping, Sweden

**Keywords:** Prehospital care, Emergency medicine, Air ambulance, Rural, Critical care, Wilderness medicine

## Abstract

**Background:**

Physician-staffed helicopter emergency services (HEMS) can provide benefit through the delivery of specialist competence and equipment to the prehospital scene and through expedient transport of critically ill patients to specialist care. This paper describes the integration of such a system in a rural Swedish county.

**Methods:**

This is a retrospective database study recording the outcomes of every emergency call centre dispatch request as well as the clinical and operational data from all completed missions during this service’s first year in operation.

**Results:**

During the study period, HEMS completed 478 missions out of which 405 (84,7%) were primary missions to prehospital settings and 73 (15,3%) were inter-hospital critical care transfers. A majority (55,3%) of primary missions occurred in the regions furthest from our hospitals, in municipalities housing only 15,6% of the county’s population. The NACA (IQR) score on primary and secondary missions was 4 (2) and 5 (1), respectively.

**Conclusions:**

This study describes the successful integration of a physician-based air ambulance service in a Scandinavian rural region. Municipalities distant from our hospitals benefitted as they now have access to early specialist intervention and expedient transport to critical hospital care. Our hospitals and most populated areas benefitted from HEMS secondary mission capability as they gained a dedicated ICU transport service that could provide specialist intensive care during rapid inter-hospital transfer.

## Background

**Fig. 1:**
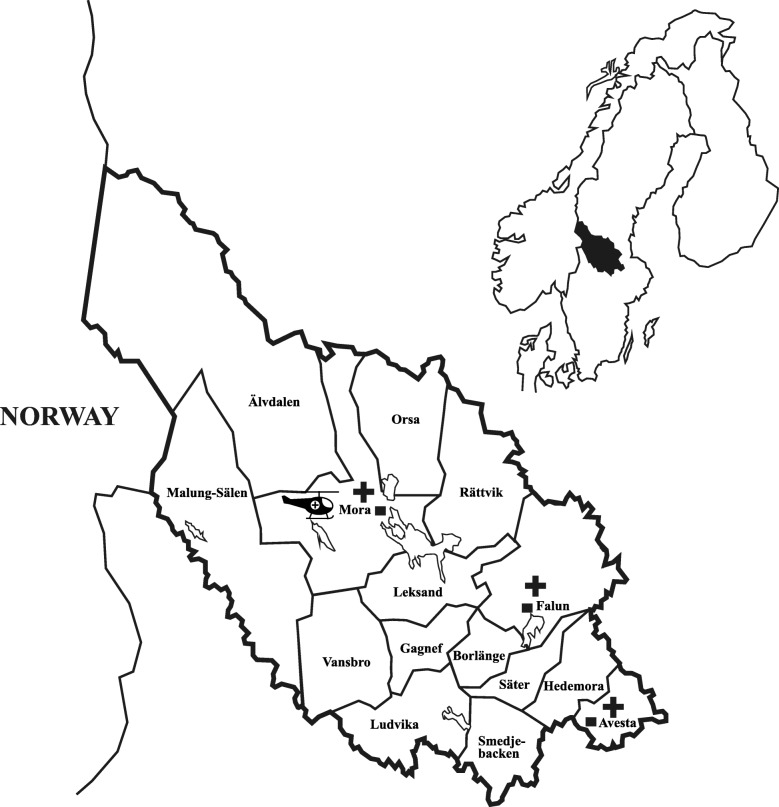
Dalarna county. Mora and Falun hospitals contain the county’s emergency departments. The helicopter is based at Mora airport, located just outside Mora. Patients requiring university hospital specialist care are transferred out of the county to Uppsala university Hospital

Dalarna is a mixed urban and rural county located in central Sweden. The region has an inland subarctic climate with cool summers and long, often cold, winters. The county has a population of 285,000 inhabitants, covers an area of 28,000 square km^2^ and is divided into 15 municipalities (Fig. 1). Lakes and rivers are prominent features in the middle, southern and eastern parts, along which the major population centres are located. Outside these, Dalarna mostly consists of vast forest areas where logging is a dominant industry. The western-most part lies within the Scandinavian mountain ranges. This alpine region houses many of Northern Europe’s most visited ski-resorts. The county annually welcomes more than 2,000,000 tourists.

Twenty-three road ambulances responding from 11 ambulance stations serve the county. Acute care is provided by three hospitals with emergency departments; Mora, Falun and Avesta hospitals. *Mora hospital* has a generalist emergency department supported by a small four-bed intensive care unit. Specialist care in obstetrics, paediatrics, vascular surgery, interventional radiology and interventional cardiology is only available at *Falun hospital*. Falun also homes the county’s major intensive care unit with eight beds. Both Mora and Falun hospitals have 24-h trauma team availability and provide most trauma and orthopaedic surgery. *Avesta hospital* has an emergency department but neither intensive care nor surgical capability. Patients requiring higher tier specialist care, including neurosurgery, thoracic surgery and stroke thrombectomy, are transferred to neighbouring Uppsala county and Uppsala University Hospital (UUH). UUH provides the region’s major trauma centre.

In 2014, Dalarna and neighbouring Värmland County started the co-owned federation Swedish Air Ambulance (SLA). This organisation was a result of the 2012 white paper report ‘Care on wings’ issued by the Swedish Association of Local Authorities and Regions [1]. The inquiry concluded that Sweden needed to build a denser network of helicopter ambulance services in order to better provide early critical care and retrieval in an era of increasing centralisation of specialist care. It was also stated that helicopters would offer increased resilience in mass casualty events. As a consequence of this report, it was decided to integrate a physician based helicopter emergency service (HEMS) into the county’s ambulance system. Helicopter base facilities were organised at Mora Airport, calculated to be the optimal location in regards to population coverage and logistics. The helicopter was dispatched for the first time in April 2016. The ensuing work aims to describe the operational and medical challenges this service faced during the first year of operations.

## Methods

In order to describe the clinical and operational characteristics, we performed a retrospective database study of every emergency call centre dispatch as well as all completed HEMS missions from 1st of July 2016 to 30th of June 2017.

### Dalarna helicopter emergency service

The service employs an Airbus H-145 helicopter platform and operates as a 24-h service with a crew consisting of one pilot, one HEMS crewmember (HCM) and one physician. Physicians are, as per SLA standards, board certified specialists in anaesthesiology and intensive care. HCM are registered specialist prehospital nurses. A helicopter technician is on 24-h duty for maintenance and technical issues. All pilots and HCM have completed night-vision imaging system training. While the helicopter remains the primary mode of dispatch, a rapid response car (RRC) is also available at the hangar facilities.

Ambulance dispatch in the region is the responsibility of the emergency medical communication center (EMCC) in Falun. As is common in the Scandinavian countries, EMCC operators *request* HEMS support when certain emergent criteria are met. It is ultimately for the physician, supported by the pilot and HCM’s assessment of weather and logistics, to decide on launch. Occasionally, HEMS will provide service to neighbouring counties on request from their dispatch centres. A request does not always result in immediate dispatch. The clinician may require further information from bystanders, callers or rescue services on scene in order to decide on launch. HEMS clinicians will stand down if they believe there is no clinical benefit from deployment or if dispatch will result in treatment delay. Occasionally, HEMS will be requested after having already accepted and launched for a prior request. The physician must then prioritise between these two requests. If a request with a valid medical indication is denied in favour of another request with higher priority, it is defined as being cancelled due to conflict of concurrency. Flight operational reasons for cancellation include technical issues or poor weather conditions. If operational issues prevent helicopter deployment, or if the patient location is in the vicinity of the base, the HEMS clinicians may respond by RRC. The majority of patients are transferred to hospital by air. The receiving Mora, Falun and UUH hospitals are equipped with elevated helipads. In some instances, road ambulance transfer is a more convenient solution, such as when the nearest appropriate hospital is nearby, cancelling any time benefit from flying. Road transport is also sometimes preferred when transporting critically ill patients who require a high degree of access and more personnel working simultaneously. Patients are transported to the nearest hospital unless that facility lacks necessary specialist capability; in which case HEMS can by-pass. In addition to primary missions to prehospital scenes, HEMS also dispatches for inter-hospital critical care transfers.

### Data abstraction and definitions

Operational data was obtained from the on-line SLA database into which the crew registers daily operational activities. Data such as the number of EMCC requests, request outcomes, mode of transport, mission timings, mission destinations, hospital destinations as well as mission types was extracted. A *completed mission* was defined as any mission that resulted in patient assessment (i.e. ‘touching the patient’). A *cancelled* mission was any mission aborted, before or after launch. Throughout this review, we use the terms ‘primary’ and ‘secondary’. A *primary* mission is an emergency response to an out-of-hospital location. A *secondary* mission is defined as an inter-hospital transfer, typically of a patient requiring a higher level of care than can be provided by the origin hospital.

We relied on five helicopter timing measurements. The *activation time* is the interval from EMCC origin call until take-off. Response time is the interval from launch to arrival at scene. Scene time is the time from arrival to the prehospital scene until departure with the patient. Transport time is the time spent transporting, by helicopter or ambulance, the patient from scene until hospital handover. Total mission time is the time from EMCC request to hospital handover. Clinical data was extracted from our paper clinical report forms as well as from hospital electronic journal systems. Severity of injury or illness was expressed using the National Advisory Committee for Aeronautics score (NACA-score) [2]. NACA scores severity from 0 (no injury nor disease) to 7 (death). Scores of five or higher are often defined as severe or critical illness or injury. The compiled clinical and operational data was entered into a database (Microsoft Excel. Version 14.5.2. Microsoft Corporation. Redmond, WA, United States.) In instances of missing data or extreme values that were unlikely to be accurate, individual clinicians were contacted and queried.

### Ethical considerations

This study was submitted to the ethical review board at the University of Uppsala for ethical vetting. We were advised that it was exempt from ethical review.

## Results

### EMCC request outcomes

**Fig. 2:**
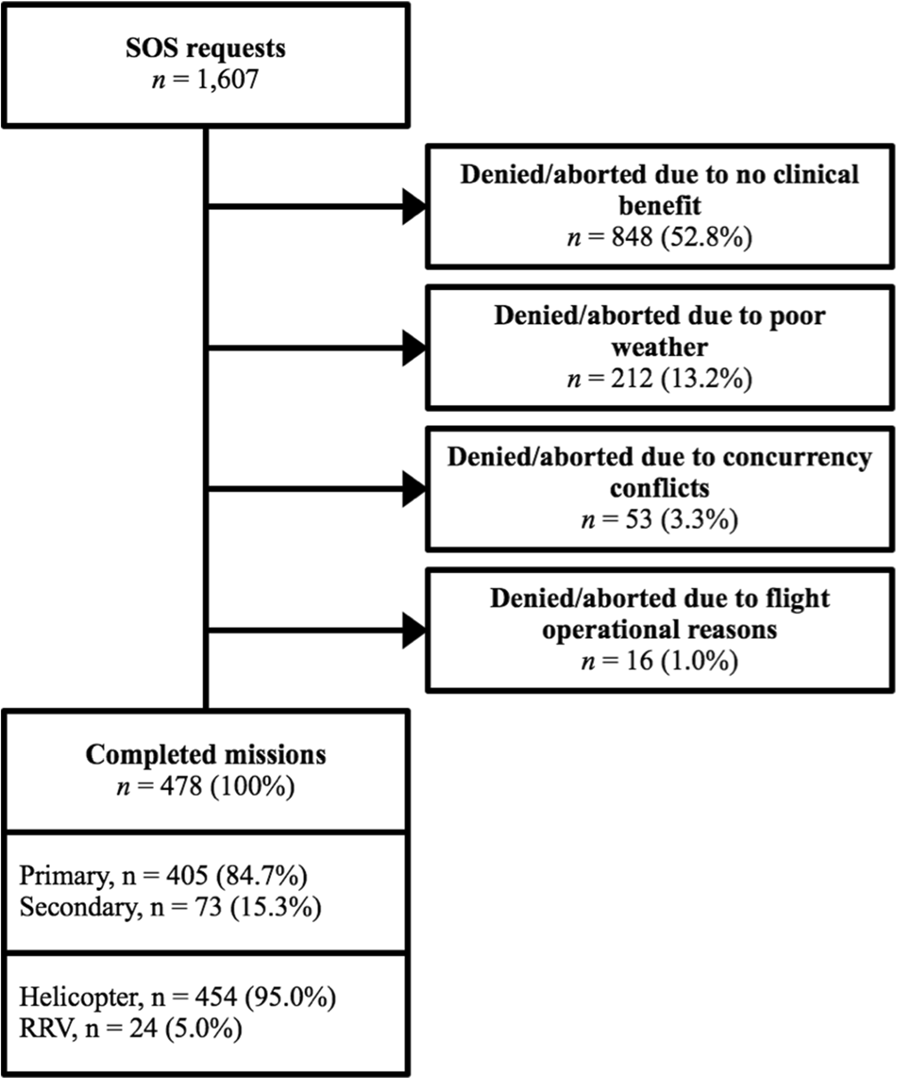
Outcomes of dispatch requests. Flowchart detailing the outcomes of all recorded emergency call center requests during the study period

**Fig. 3:**
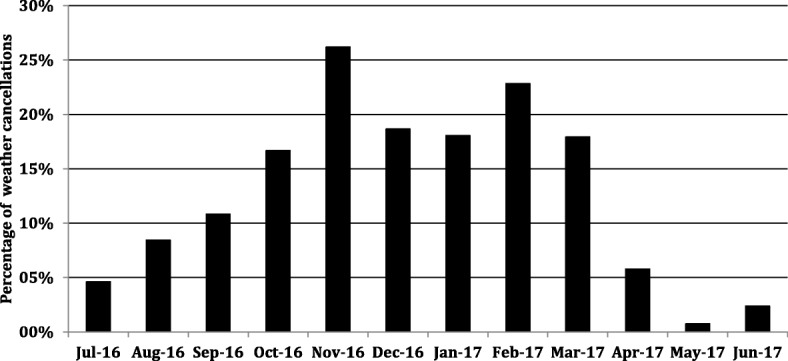
The impact of request cancellation due to weather. Each column represents the monthly percentages of all emergency call center requests that were cancelled for poor weather and visibility. The rate of weather cancellation increased during the winter months

During the 12-month study period the EMCC requested HEMS attendance on 1607 occasions (Fig. 2). Of these, 52.8% were cancelled before dispatch or aborted after launch as there was no apparent clinical benefit. In 13.2% of EMCC requests, poor weather was registered as the primary reason for cancellation. Weather cancellations displayed great seasonal variation (Fig. 3). In July 2016, only 4.6% of missions were cancelled due to weather. Weather then deteriorated in the autumn months. In November, 26% of requests were cancelled due to weather after which conditions gradually improved. 3.3% of requests were denied due conflicts of concurrency (Fig. 2). Only 1% of requests were denied for flight operational reasons. Four hundred seventy-eight requests resulted in patient management. 84.7% of completed missions were primary missions. Completed missions alone do not represent HEMS flight activity as many cancellations were decided upon while en-route. During the study period HEMS launched a total of 807 times.

### Primary mission operational characteristics

**Fig. 4:**
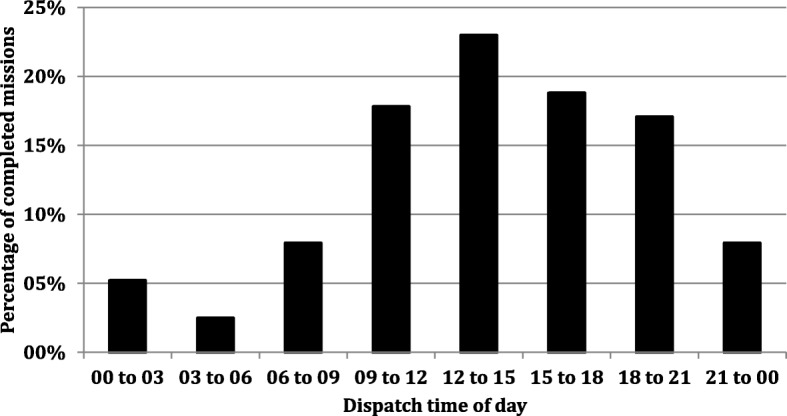
The temporal distribution of completed missions. Each bar represents the percentage of completed missions that HEMS launched for during that time interval

Primary mission dispatch displayed a distinct diurnal variation (Fig. 4). As is evident, significantly more missions were carried out during daylight hours. The majority of missions were undertaken in the scarcely populated westernmost municipalities where distances to hospitals were the longest (Table 1, Fig. 1). Of patient encounters, 55.3% occurred in Malung-Sälen, Älvdalen, Mora and Vansbro despite how these municipalities only house 15.6% of the county’s population. This dominance can only partly be ascribed to the seasonal influx of ski tourists. In our data from the 6 months (July 1st to January 1st), before the start of the ski high season, these four municipalities still account for 47,6% of missions. The vast majority (95.6%) of HEMS dispatches were helicopter launches. Only 4,4% of primary missions were carried out using the RRC. 58.8% of patients were transported to hospital by helicopter (Table 1). In 30.1% of patients, road ambulance was considered the most appropriate mode of transport. Eleven percent of patients were not transported at all; occasionally because of over-triage or improvement, but mostly after life was pronounced extinct on scene.

**Table 1:** Missions destinations and modes of transport

	12 months		6 months			
	*N*	(%)	*N*	(%)	Population	% of total population
Destination municipality						
Total	405	100,0%	186	100,00%	284,700	100%
Malung-Sälen	75	18,5%	28	15,1%	10,100	3,5%
Älvdalen	61	15,1%	26	14,0%	7000	2,5%
Mora	54	13,3%	19	10,2%	20,300	7,1%
Vansbro	34	8,4%	19	10,2%	6900	2,4%
Ludvika	31	7,7%	17	9,1%	26,900	9,5%
Rättvik	26	6,4%	15	8,1%	10,900	3,8%
Out of county	23	5,7%	15	8,1%	N/A	N/A
Leksand	21	5,2%	14	7,5%	15,500	5,5%
Gagnef	18	4,4%	9	4,8%	10,200	3,6%
Orsa	18	4,4%	6	3,2%	6900	2,4%
Falun	14	3,5%	6	3,2%	57,700	20,3%
Hedermora	9	2,2%	4	2,2%	15,500	5,4%
Säter	8	2,0%	3	1,6%	11,100	3,9%
Smedjebacken	6	1,5%	2	1,1%	10,900	3,8%
Avesta	4	1,0%	2	1,1%	23,200	8,1%
Borlänge	3	0,7%	1	0,5%	51,600	18,1%
Transport to scene	*N*	(%)	Destination hospital		*N*	(%)
Total	405	100,0%	Total		360	100,00%
Helicopter	387	95,6%	Falun		176	48,9%
Car	18	4,4%	Mora		157	43,6%
Transport from scene	*N*	(%)	UUH		14	3,9%
Total	405	100,0%	Other		13	3,6%
Helicopter	238	58,8%				
Car	122	30,1%				
Not transported	45	11,1%				

The destination municipalities of primary missions during the study period at 12 and 6 months. Out of county destinations were neighboring counties or cross-border locations in Norway. The bottom left table details the modes of transport to and from scene, respectively. The bottom right table describes hospital destinations

Our median (IQR) helicopter activation time was 9 (6) minutes. Response time was 24 (17) minutes. Scene time was 20 (17) minutes. Our transport time, at 40 (19) minutes, was longer than the response time. The discrepancy between response and transport times is partly explained by how HEMS often by-passed Mora hospital in favour of Falun hospital, UUH or other out-of-county hospitals. Forty-eight percent of patients were handed over to Falun hospital vs. 43,6% to Mora (Table 1). Fourteen primary mission patients were transferred directly to UUH. Our median (IQR) total mission time was 90 (42) minutes. Importantly, this measurement does not include time subsequently needed to return to the hangar, refuel, restock or to complete documentation. Long or extreme mission timings were recorded for out-of-county missions or cross-border activity in Norway as well as for direct or secondary transfers to UUH.

### Primary missions clinical characteristics

As is summarised in Table 2, in 405 primary missions, trauma was the dominant dispatch condition at 40.0%, followed by chest pain (11.4%) and cardiac arrest (10.9%). The median age was 46 years and male gender was the more prevalent at 66.4%. Fifty-six patients (13.8%) were 12 years or younger, of whom 21 were infants (0–2 years). The median NACA (IQR) score was 4 (2). While a detailed description of clinical subgroups is outside the scope of this manuscript, a brief narrative is helpful to describe our medical challenges.

**Table 2:** Overview of primary and secondary missions

SOS request criterium	*N* (%)		NACA, median (IQR)		Age in yrs., median (Range)		Male gender *N* (%)	
Primary missions	405	100,0%	4	2	46	0 to 94	269	66,4%
Trauma	162	40,0%	3	1	38	0 to 90	113	69,8%
Chest pain	46	11,4%	4	1	63	31 to 91	37	80,4%
Cardiac arrest	44	10,9%	6	0	66	0 to 89	32	72,7%
Dyspnoea	31	7,7%	4	2	16	0 to 94	15	48,4%
Stroke	24	5,9%	3	1	72	11 to 86	13	54,2%
Reduced LOC	22	5,4%	4	2	59	0 to 92	12	54,5%
Intoxication	19	4,7%	3	2	29	3 to 52	15	78,9%
Seizures	18	4,4%	3	1	20	0 to 88	10	55,6%
Other	14	3,5%	3	2	34	0 to 81	6	42,9%
Anaphylaxis	10	2,5%	3	1	32	0 to 75	7	70,0%
Acute abdominal pain	6	1,5%	3	1	58	8 to 72	6	100,0%
Pregnancy	6	1,5%	3	0	22	16 to 26	0	0,0%
Drowning	3	0,7%	6	1	65	7 to 75	3	100,0%
Secondary Missions	73	100,0%	5	1	53	0 to 86	42	57,5%

Overview of all completed missions. Severity is expressed using median NACA with interquartile ranges (*IQR*). Age is presented as median age with absolute range in years

*Traumatic injury* included severe polytrauma, severe maxillo-facial trauma, head injury, chest trauma, abdominal trauma as well as pelvic and long bone fractures. Trauma interventions included endotracheal intubation, thoracostomies, external haemostasis, repositioning and splinting of fractures, invasive blood pressure monitoring as well as neuroprotective care. Notably, HEMS attended one serious mass casualty incident. In April 2017, a bus carrying school children turned over in a very remote location with devastating consequences. Three children were pronounced dead on scene, five were defined as severe or critically injured and numerous others were treated for lesser injuries. In this incident, the HEMS clinicians performed life-saving specialist interventions, followed by direct transfer to the UUH major trauma centre for urgent neurosurgical intervention.

*Non-traumatic critical illness* included obstructive lung disease, pneumonia, acute cardiac failure, arrhythmia, ST-elevation myocardial infarction, cardiac arrest, ruptured abdominal aneurysm, intoxication, seizures, meningitis, septic shock as well as ischaemic and haemorrhagic stroke. Interventions included tracheal intubation, non-invasive positive pressure ventilation, inhalational therapy, vaso- and cardioactive infusions, advanced life support, post ROSC care, blood cultures, antibiotics, seizure management, invasive blood pressure monitoring, and intracranial pressure management. Sixty-nine primary patients had prehospital endotracheal intubation of which 40 were drug-assisted. All (100%) of attempted tracheal intubations were successful on the first or second attempts. An intubation attempt is in our database defined as starting when the laryngoscope passes the patients front teeth.

### Secondary mission characteristics

During the study period HEMS completed 73 secondary missions. With the exception of the cardiac arrest patients, this group had the highest median NACA (IQR) score of 5 (1). The vast majority were helicopter transfers from Mora or Falun to UUH of patients requiring specialist university hospital care in the form of cardiothoracic surgery and neurosurgery. During the study period, transfers for stroke thrombectomy became increasingly common.

## Discussion

**Fig. 5:**
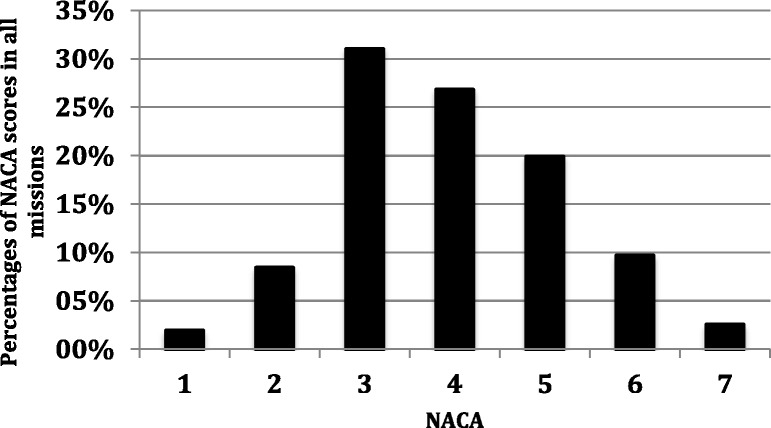
NACA proportions. The proportions of NACA scores in all completed primary (*n* = 405) and secondary (*n* = 73) missions

Integrating physician-based HEMS into a rural ambulance system can be beneficial. Specialist competence and equipment can be rapidly delivered to the prehospital scene. The helicopter can provide expedient transport to definitive care. Patients in inaccessible locations can be reached [3]. In addition, secondary mission capability allows intensive care to proceed without interruption during rapid transfers between hospitals. This paper reports the implementation of HEMS in a large rural Scandinavian region. During the study period HEMS completed 478 missions out of which 405 were primary missions. This primary mission rate of 14.5 missions per 10.000 registered inhabitants, as well as the primary to secondary mission ratio of 84,7% is similar to other Scandinavian regions [4, 5]. Our primary mission median (IQR) NACA score of 4 (2) compares well to other Scandinavian HEMS and we have no immediate concerns of unacceptable overtriage [5, 6]. Our clinicians frequently managed severely ill or injured patients, often performing highly invasive and critical interventions. Almost one third of all patients had NACA scores of 5 to 7, signifying critical illness or injury (Fig. 5). Roughly half of patient encounters occurred in the scarcely populated western mountain municipalities. Fewer missions were completed in the densely populated central and eastern regions. Thus, the helicopter acted as an equaliser as rural populations gained access to early critical care and rapid hospital transport. Patients were in several instances transferred directly from scene to out-of-county university hospital specialist care. This equalising effect has been documented by numerous authors and was one of the desired goals of HEMS establishment and the ‘Care on Wings’ white paper [6–11]. While the rural areas profited the most from helicopter primary mission coverage, central area ambulance services benefited from HEMS performing 73 inter-hospital intensive care transfers. This secondary mission capability liberated ground ambulance as well as hospital resources and spared the county of expenses from contracting out-of-county air retrieval services. With HEMS, the region gained a dedicated ICU transport service that could provide specialist care during rapid inter-hospital transfer with minimal exposure to time between hospitals.

While we are satisfied with progress during our first year, we intend to closely monitor future developments. It is possible that liberal dispatch conditions in association with organisational inexperience have generated overtriage in dispatches and missions. Nevertheless, if our service follows the pattern of other newly established Scandinavian HEMS, our caseload is going to increase in the coming years. During their first 2 years of full operations, the Danish Air Ambulance saw an increase in the number of completed missions by roughly 40% [12]. Similarly, most Norwegian HEMS bases, if not all, have increased their caseload significantly during the years following their inception [11, 13]. The accumulation of experience amongst dispatchers, pilots and medical crew as well as active improvement processes are likely to drive such developments.

HEMS overall cancellation rate of 70% was comparatively high. Without detailed knowledge of dispatch criteria, geography, activation thresholds and regional weather patterns, request outcomes are difficult to compare. Nevertheless, in a Norwegian study, Østerås et al. reported a much lower cancellation rate at 38% [5]. The majority of HEMS cancellations were due to lack of apparent clinical benefit. In these requests the specialist competencies of the HEMS team would not add any clinical value to the patient, nor would the patient benefit from faster transport to hospital.

This high rate is explained by several factors. It is possible that our current dispatch criteria are too liberal and generate excessive overtriage in requests. Furthermore, EMCC operators frequently make dispatch requests based on limited information, resulting in subsequent cancellations as progressively more information becomes available. Unfortunately, robust evidence-based HEMS tasking criteria remain elusive. This is particularly true for services that often deploy to non-trauma cases [14–16]. We intend to keep calibrating our dispatch conditions in order to optimise triaging. The active design of, and adherence to, HEMS-specific dispatch criteria can result in improved accuracy and reduced cost [17–20]. Many requests were cancelled due to EMCC operators being instructed to not discriminate against location when requesting HEMS. Requests from central areas close to hospitals were often denied as high levels of care were achieved faster if ambulance conveyed without waiting for HEMS. This difficulty in providing benefit in the most populated regions is partly the consequence of the helicopter being stationed in Mora. However, a more central location would diminish the desired effect of improved coverage in the western rural regions. In any case, with increased dispatcher and crew experience, we gain a better understanding of infrastructure and geography. We will become better at delineating areas where we can provide intervention without delaying the chain of care.

We cannot ascertain the true impact of weather on medically valid missions. In instances were poor weather clearly would prevent dispatch; clinicians tended not to further interrogate EMCC about the medical indication. Still, it is clear that poor weather is a major limiting factor for us, as well as for other systems operating in climates similar to ours [5, 21]. Overall, with great seasonal variation, 13.2% of HEMS missions were cancelled due to weather. Particularly troublesome was how a majority of primary mission destinations were in the western mountain and highland ranges allowing little opportunity to salvage missions by navigating to lower altitudes and more accommodating weather. Other services have documented weather cancellation rates in the range of 5.1 to 30% [5, 21]. Again, the literature is difficult to compare due to differences in topography, population distribution, climate and the minimal weather requirements of individual operators. Our incidence of weather cancellations increased during evenings and nights. This was not only due to improving weather during daylight hours. Our pilots are also required to operate in accordance to stricter weather and visibility requirements after nightfall. Other authors have documented this. Østerås et al. reported that weather cancellations increased roughly threefold at night [5]. This pattern of weather cancellations amplified the diurnal pattern of completed missions that is otherwise explained by the circadian occurrence of several emergent conditions (Fig. 4) [22–25]. Nevertheless, we expect cancellations due to weather to decrease as our flight crews become more familiar and confident with our topography and regional weather patterns. The increasing implementation of technology such as GPS approach systems and weather webcam systems may increase efficacy [26].

As we summarise our experiences, we find that operating in a region such as ours comes with particular challenges. As reported by other Scandinavian HEMS, our clinicians frequently performed critical and complex interventions in severely ill patients suffering from a wide range of illness and injury [27–29]. HEMS activity in the distant western regions is reflected in our, even by Scandinavian standards, long flight times and distances [4]. This represents another marker of rural HEMS; clinicians are frequently forced to manage critically ill patients for prolonged periods of time [10]. With our subarctic climate; situations were often made more complex as they had to be managed under exposure to poor weather, sub-zero temperatures with snow and ice.

We believe maintaining current expertise in advanced airway management, anaesthetics and intensive care is key to performing these duties in a safe manner. As suggested by other authors, the Scandinavian anaesthesiologists, who have dual training in anaesthesia and critical care, working in teams with highly trained specialist aeromedical nurses are well suited for this role [30–33]. Nevertheless, it must be acknowledged that many Scandinavian HEMS operate in scarcely populated regions. As some critical procedures and assessments are performed infrequently, significant on-going in-hospital duty as well as targeted training is of crucial importance in order to maintain proficiency and safety [29, 34].

### Limitations

Our study has several limitations. It is of a retrospective nature and cohort data is based on self-reporting by clinicians employed by the helicopter service. Data was collected during this service’s first year in service. It is likely that the outcomes reported above will change as our institutional and individual experience increase. Factors that are particular to our county such as geography, weather, population, ambulance cover and dispatch procedures may limit generalisability to other regions.

## Conclusion

This work describes the operational and clinical characteristics of a newly organised rural HEMS that is staffed by anaesthesiologists and specialist nurses. Our clinicians have provided specialist intervention in a wide range of critical illness or injury. Our population, especially the rural areas, has benefited from early specialist intervention and expedient helicopter transport to specialist care. We have identified areas that require improvement and close monitoring. As with any HEMS, our system is shaped by regional geography, population patterns and climate.

## Abbreviations

HCM: HEMS Crewmember
HEMS: Helicopter Emergency Medical Service
NACA: National Advisory Committee for Aeronautics
RRC: Rapid Response Car
